# Gastroblastoma without GLI1 and EWSR1 gene breaks

**DOI:** 10.1186/s12957-023-03159-7

**Published:** 2023-09-01

**Authors:** Can Gong, Junyi Xu, Shuye Qiao, Xuemei Zhang, Min Yi

**Affiliations:** 1 Department of Clinical Pathology, Liuzhou Workers’ Hospital, Guangxi, China; 2 Department of Gastrointestinal Surgery, Liuzhou Workers’ Hospital, Guangxi, China

**Keywords:** Gastroblastoma, Biphasic differentiation, Clinicopathologic feature, GLI1, EWSR1

## Abstract

**Objective:**

To report a rare gastroblastoma; discuss its clinical features, histopathological morphology, diagnosis, differential diagnosis, treatment, and prognosis; and so as to improve the understanding on this disease and provide reference for its diagnosis, treatment, and prognosis.

**Methods:**

The diagnosis and treatment, imaging examination, pathological, and genetic data of a 19-year-old young female patient with gastroblastoma were analyzed retrospectively, and the relevant literature was reviewed and summarized.

**Results:**

The patient was found to have a “gastrointestinal stromal tumor” for 3 days by physical examination in another hospital. Abdominal CT and MRI considered “solid pseudopapilloma of pancreas” and clinically planned to perform “radical pancreatoduodenectomy.” During the operation, the tumor was observed to bulge from the posterior wall of the gastric antrum, and the root was located in the gastric antrum, so it was changed to “partial gastrectomy + Ronx-y gastrojejunal anastomosis.” The postoperative pathology showed that the tumor was bi-differentiated between gastric epithelium and mesenchymal. Combined with the results of IHC and the opinions of several consultation units, the diagnosis of gastric blastoma (low-grade malignancy) was supported. However, the fracture rearrangement of GLI1 and EWSR1 genes was not detected by FISH. After 19 months of follow-up, no signs of tumor recurrence and metastasis were found.

**Conclusion:**

Combined with existing literature reports, gastroblastoma occurs in young people, equally in men and women, and tends to occur in the gastric antrum. The biological behavior of the tumor tends to be inert, and the prognosis of most cases is good. Postoperative pathology and IHC are reliable methods for the diagnosis of gastric blastoma, and surgical resection of the lesion is the preferred treatment.

## Clinical features

**Fig. 1:**
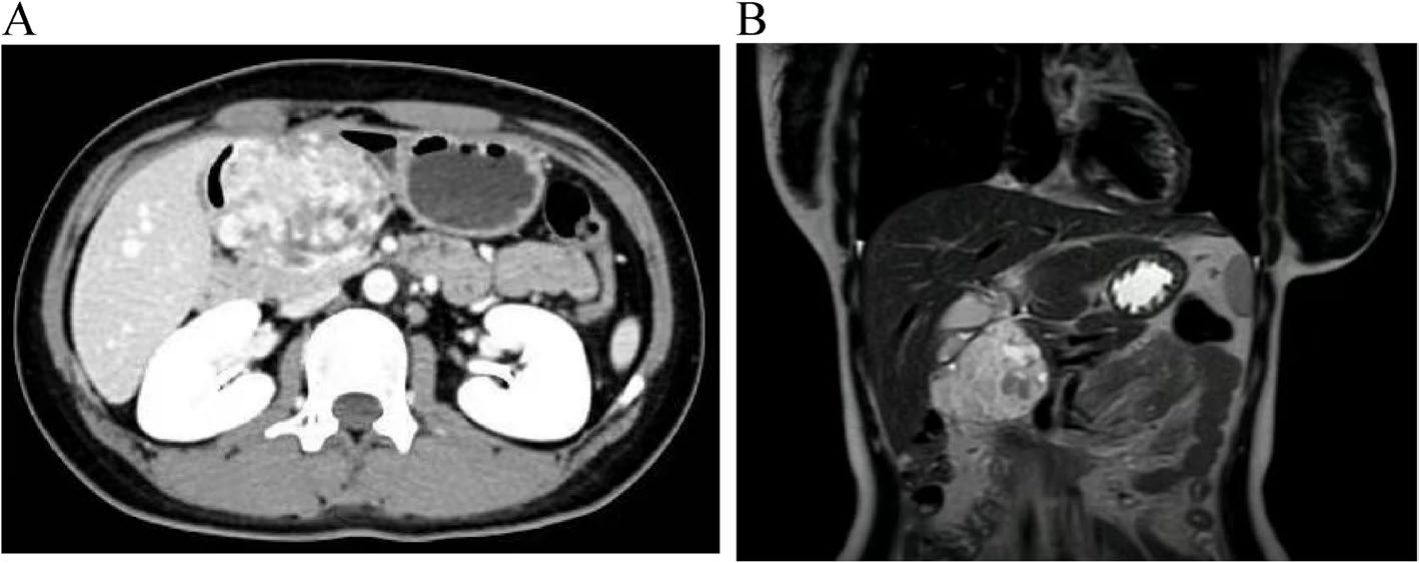
Imaging examination **A** Abdominal CT shows a mass in the pancreatic head area, the boundary was clear, and the boundary between the lesion and the pancreatic head was not clear. **B** Abdominal MRI showed a solid mass in the pancreatic head, with clear boundary, and internal necrosis and sac

The 19-year-old female patient was admitted on August 12, 2021, due to “3 days of gastrointestinal stromal tumor was found during physical examination in an external hospital.” In the past half a year, the appetite was poor and the weight decreased by 6 kg. Physical examination showed that a tumor could be touched on the upper part of the umbilical cord, about 10 cm × 10 cm in size, hard quality, blurred edges, general activity, no tenderness, or rebound pain in the whole abdomen. Laboratory examination showed no obvious abnormalities in routine blood tests, liver and renal function, coagulation function, and pancreatic function; tumor markers (AFP, CA125, CA19-9, CA15-3, CEA, FERR, CA50, CA242, SCCA, CA72-4) were within the normal range. Gastroduodenoscopy shows compression changes in the gastric antrum. Abdominal B-ultrasound shows pancreatic head occupation, SPN. The abdominal CT showed the pancreatic head area, clear boundary, unclear boundary between the lesion and the pancreatic head, obvious compression of the stomach and duodenum, and the internal septation with multiple cysts. “Pancreatic solid false papilloma” was considered (Fig. 1A). The abdominal MRI showed a solid mass in the pancreatic head with clear boundary, about 8.1 cm × 6.9 cm × 4.6 cm in size, necrosis, and cystic changes, which was considered as “Pancreatic solid false papilloma” (Fig. 1B), a clinical plan to perform “radical pancreatoduodenectomy.” During the operation, the tumor was observed to bulge from the posterior wall of the gastric antrum, and the root was located in the gastric antrum, pylorus dorsal, to the pancreatic head compression. The tumor has clear boundaries and complete envelope, no invasion or adhesion with surrounding organs, no enlarged lymph nodes in the abdominal cavity, and no tumor in the pancreas. Therefore, it was changed to “partial gastrectomy + Ronx-y gastrojejunal anastomosis.” The operation lasted for 3 h, the operation course was smooth; the anesthesia was satisfactory; the patient’s vital signs were stable; the intraoperative bleeding was about 30 ml, without blood transfusion; and the surgical infusion volume was 1700 mL. On the 5th postoperative day, the blood routine + CRP showed leukocyte count: 26.07 × 10^9/L, and the other indicators were not special, so anti-infection treatment was immediately given. Physical examination showed no fever and sputum cough. After 14 days in hospital, the patient had a general condition, no abdominal distension, abdominal pain, no chest tightness, shortness of breath, and other discomfort. The abdominal incision II/Grade A was healed, and stitches were removed and discharged successfully. The patient was followed up for 19 months and showed no signs of tumor recurrence or metastasis.

## Pathological findings

### Pathological examination

Part of the gastric tissue submitted for inspection and a relatively clear mass was found on the serosal surface of the gastric wall. The size of which was about 8.0 cm × 6.9 cm × 4.5 cm. On the cut surface, the tumor was solid, white–gray and red in color, and partially cystic with areas of necrosis and mucoid attachment. No enlarged lymph nodes were found during the operation, so no lymph node dissection was performed.

### Microscopy

**Fig. 2:**
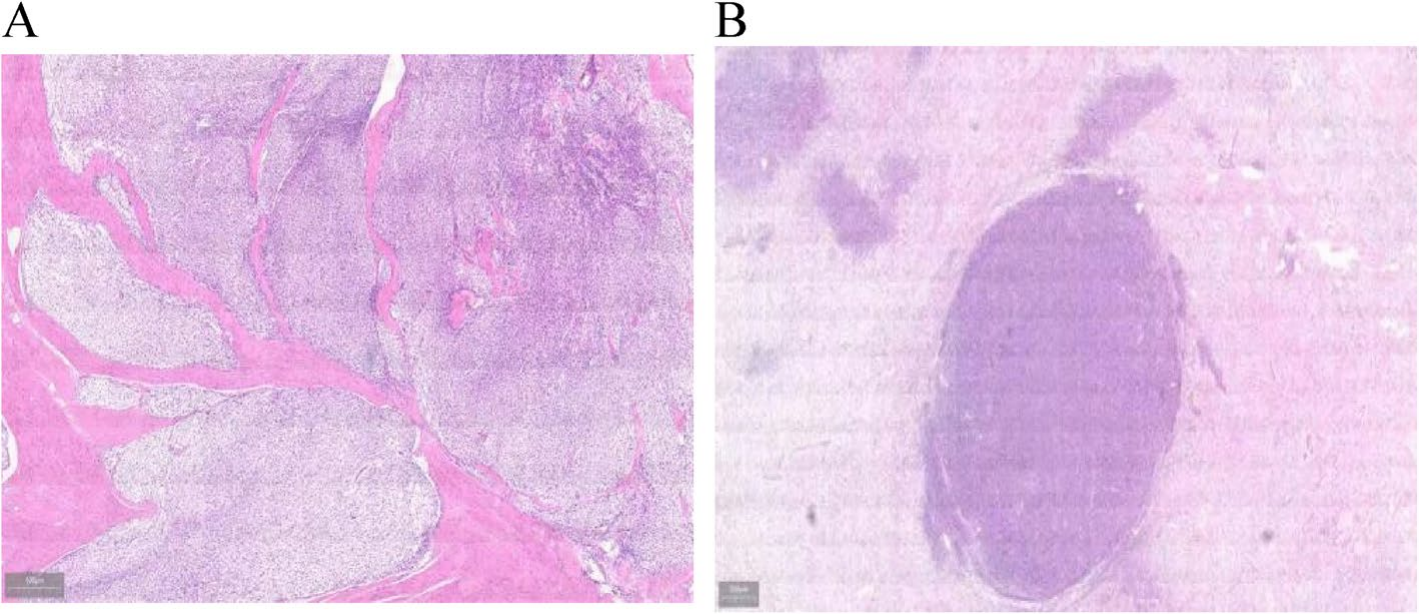
Histological morphology of gastroblastoma **A** Tumor cells grow in the mucosa, submucosa and muscularis propria, and some areas are separated into nodulules and nests by smooth muscle bundles. **B** Tumor is composed of epithelium and mesenchyma, which are intermixed, but the boundaries are relatively well-defined

The tumor grew infiltratively in the submucosa, muscle layer, and serosa layer, locally infiltrating into the mucosal lamina propria, and parts of the tumor were separated into nodular and nested by smooth muscle bundles (Fig. 2A). The tumor was composed of epithelium and mesenchyma, among which the mesenchyma accounted for about 90%. The epithelial components were scattered foci and mixed with the interstitial lobe, but the boundary was still clear (Fig. 2B). Epithelioid pattern, with mild cell morphology and fine nucleoli; mesenchyme is composed of sparse and dense areas, spindle tumor cells, with mild atypia, myoid degeneration in some stromal areas, and nuclear division < 5/50HPF.

## Immunophenotype

**Fig. 3:**
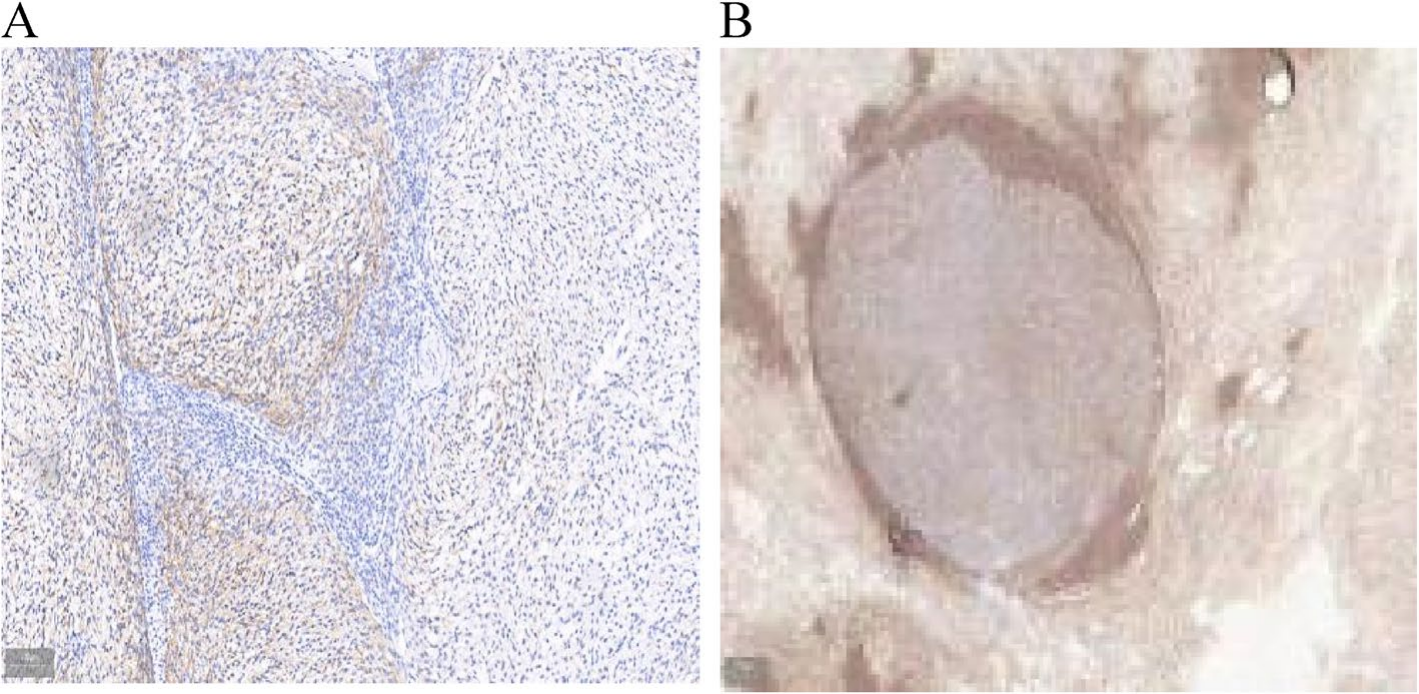
Immunohistochemistry in gastroblastoma **A** EMA was strongly positive in the epithelial area and negative in the surrounding mesenchymal component. **B** CD56 was strongly positive in the mesenchymal component and epithelial area

Epithelioid regional tumor cells expressed EMA, CKpan, CD10, and CD56 (Fig. 3A), and mesenchymal component tumor cells expressed vimentin, CD10, and CD56 (Fig. 3B). Tumor cells in the epithelial and mesenchymal regions DOG-1, CD34, S-100, SOX-10, STAT 6, SMA, desmin, Syn, and CgA were negative; SDHB-positive expression; Ki-67 proliferation index was 3%.

## Molecular pathology

**Fig. 4:**
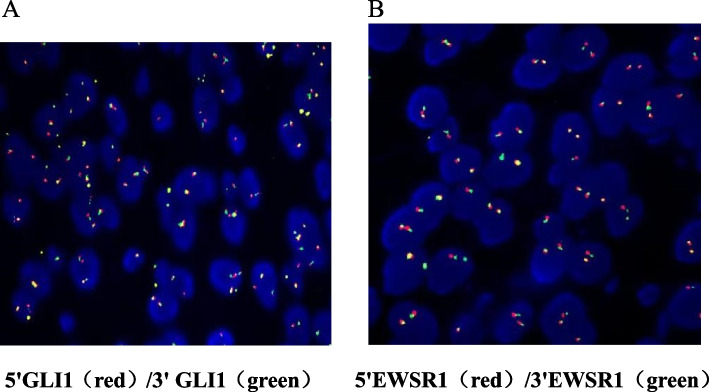
Fluorescence in situ hybridization (FISH) in gastroblastoma **A** No GLI1 gene break rearrangement was detected. **B** No EWSR1 gene break rearrangement was detected

The fluorescence in situ hybridization (FISH) test of the tumor tissue showed no broken rearrangement of the GLI1 gene (Fig. 4A) and that of the EWSR1 gene in the patient’s tumor (Fig. 4B).

## Pathologic diagnosis

Combined with the histological morphology of the tumor components, the immunohistochemical results and the opinions of several consultation units, and all supported the diagnosis of gastric blastoma (low-grade malignancy).

## Follow-up

After partial gastrectomy, no specific treatment was given, and no recurrence or metastasis was observed until March 5, 2023.

## Discussion

Gastroblastoma is an extremely rare biphasic neoplasm firstly reported by Miettinen et al. in 2009 [1]. The pathogenesis of this tumor is still unknown, but some scholars believe that it develops from a kind of totipotent stem cells [2]. To date, a total of 17 cases [1, 3–16] have been reported in domestic and foreign literature, including 13 in English literature [1, 3–12] and 4 in Chinese literature [13–16]. The clinical data and follow-up information have been summarized in Table 1. The WHO (2019) 5th edition of the digestive system tumor classification included gastric blastoma for the first time and was placed under the malignant epithelial tumor category of the stomach, with ICD-O code of 1, suggesting that it has low malignant potential [2].

**Table 1:** Clinicopathologic features of 17 cases of gastroblastomas

Miettnen et al. [1]	30	man	Anti-force, anemia	sinuses ventriculi	15 × 12	Gastrosinus resection + postoperative radiotherapy	For 14 years, with disease-free survival
Miettnen et al. [1]	27	woman	Abdominal pain, and an abdominal mass	Large curved stomach	6 × 4 × 3.5	Partial gastrectomy	5 years:disease-free survival
Miettnen et al. [1]	19	man	Abdominal pain, and an abdominal mass	Large curved stomach	5 × 4 × 2.5	The stomach was excised in large parts	At 3.5 years, with disease-free survival
Pinto et al. [3]	53	woman	indigestion	Large curved stomach sinus	2.3	Partial gastrectomy	1.5 years, disease-free survival
Centonze et al. [4]	43	woman	enterorrhagid	sinuses ventriculi	5.3	Partial gastrectomy	And 8.4 years, with disease-free survival
Castri F et al. [5]	79	man	abdominal mass	sinuses ventriculi	3	Partial gastrectomy	not clear
Toumi et al. [6]	29	woman	Upper abdominal pain, vomiting blood	Large curved stomach	7	Partial gastrectomy + splenectomy	Local recurrence in situ after 6 months
Na Zheng et al. [7]	12	man	bloody stool	not clear	7	Subtotal gastric resection	For 8 months, with disease-free survival
Yangyang Ma et al. [8]	12	man	Hemochezia, and abdominal mass	sinuses ventriculi	4.5 × 2.5 × 2.5	The stomach was excised in large parts	For 8 months, with disease-free survival
Teresa Femand-ez et al. [9]	19	woman	Abdominal pain, radiating to the back, abdominal mass	sinuses ventriculi	10.5	A distal gastrectomy was performed	At 20 months, with disease-free survival
Wey et al. [10]	28	man	Constipation, and an abdominal mass	Far stomach	3.8 × 3.3 × 2.5	Preoperative chemotherapy + partial gastrectomy	Liver, lymph node, retroperitoneal and bladder metastases; Three months after surgery, the clinical condition is good
Shin et al. [11]	9	man	Abdominal pain, and an abdominal mass	Gastric bin	9 × 6.5	Pargastric antrum + pylorus removed	At 9 months, with disease-free survival
Koo et al. [12]	17	man	Wiskott-Aldrich syndrome	fundus ventriculi	6.3	Partial gastrectomy	23 months: disease-free survival
Zhu na et al. [13]	65	woman	Physical examination of gastric space	Large curved stomach body	1.3 × 1 × 0.9	Full-thickness resection of the gastric wall (EPTR)	For 4 years, with disease-free survival
Long Wei guo et al. [14]	53	woman	The upper abdomen is dull and painful	The junction of gastric antrum and gastric body	5.0 × 4.2 × 4.0	Partial gastrectomy	At 14 months, with disease-free survival
Chen Si min et al. [15]	51	woman	Black stool, dizziness, black dark	sinuses ventriculi	2.8 × 1.8 × 1.5	Partial gastrectomy	At 9 months, with disease-free survival
Chen Qi et al. [16]	43	man	Reverse the acid and make a black stool	sinuses ventriculi	5.5 × 5.3	Partial gastrectomy	2 years: disease-free survival
Our case	19	woman	Loss of appetite, and lost body weight	sinuses ventriculi	8.1 × 6.9 × 4.6	Partial gastrectomy	At 19 months, with disease-free survival

Gastroblastoma usually occurs in young patients and occasionally in the elderly. The ages ranged from 9 to 79 years, the average age is 35 years old, and the proportion of men and women is close to 1:1. Gastrlastoma was most commonly found in the gastric antrum, followed by the greater curvature of the gastric body, with a maximum diameter of 1.3 ~ 15 cm and an average of 5.7 cm. In most of the reported cases, patients often have no obvious specific clinical manifestations and symptoms, most of them are abdominal pain, abdominal space, hematochezia, anemia, and fatigue. This 19-year-old young female patient, with no obvious clinical symptoms, was initially diagnosed as “gastrointestinal stromal tumor” by physical examination and was admitted for CT and MRI, and both of which showed a solid mass in the pancreatic head with clear boundary, necrosis, and cystic change, which was considered as “pancreatic solid false papilloma”. Clinically, “radical pancreaticoduo-denectomy” was planned. During the operation, the tumor was swollen from the posterior wall of the antrum, with exogenous type, and the root was located in the dorsal side of the antrum and pylorus, and compressed to the pancreatic head, that no mass in the pancreas, so it was changed to “partial gastrectomy”.

Gastroblastomas is generally exophytic growth, showing relatively large multinodular or lobulated, often solid, can also be cystic or ulcerated, with clear boundaries, and the maximum diameter of the tumor is about 1.3 ~ 15.0 cm. The cross-section of the tumor is varied and can be colorful, mottled, or hemorrhagic. Microscopically, the tumor showed infiltrative growth and could involve the whole layer of the gastric wall or be confined to the serosal layer. The tumor mainly consists of two components with different proportions of epithelioid cells and spindle cells. The epithelioid cells can be arranged in nest clumps, cables, tufts, or ducts, in the lumen with or without eosrotropic secretion, with mild cell morphology and partial fine nucleoli. The region of the fusiform cells is bundle, sheet, can also be loose network, vortex, cell morphology is more consistent, oval or short spindle, less cytoplasm, and the nucleus has no obvious atypia. Stroma can be accompanied by sclerosis or myxoid degeneration, and in some cases, pulmonary edema-like structure; multinucleated giant cells, erythrocyte extravasation, and occasionally necrosis or calcification may occur [8, 9]. Although the epithelial and mesenchymal components are often fused, the boundary of the two components is still clear. Most of the mitotic images were 0–5/50HPF, and only one case was as high as 30/50HPF. In this case, the main body of the tumor was located in the submucosa, muscle layer, and serosal layer, and it locally invaded the mucous lamina propria. Some regions were separated by smooth muscle bundles into nodules and nests, the cell morphology was mild, most of them were fusiform, multiple foci were epithelioid, small nucleolus could be seen, mitotic images were rare, and some regions of interstitial mucoid degeneration.

Immunohistochemical staining showed that epithelial regional tumor cells CK (AE1/AE3), CK7, CAM5.2, and EMA were all positive, expressing CD56 and CD10 to varying degrees, and some cases expressed CK8 and CK18, but CK20 was usually negative; mesenchymal regional tumor cells were positive for Vimentin, CD56, and CD10 and expressed CD99 to varying degrees. In some cases, desmin was patchy positive in stromal area or CD117 positive in epithelial area, and Ki-67 proliferative index was low (< 5%). Tumor cells in the epithelial and mesenchymal regions were negative for DOG-1, CD34, S-100, SOX-10, STAT 6, SMA, C-Kit, Calretinin, Syn, CgA, TTF-1, ER, and PR. The immunohistochemical results of this case were consistent with the reported gastroblastoma.

In terms of cellular and molecular genetics, six of the reported cases confirmed the presence of the MALAT1-GLI1 fusion gene [5, 15, 17], and Graham et al. [17] first confirmed these results at the transcription and genomic levels by reverse transcription-polymerase chain reaction (RT-PCR) and fluorescence in situ hybridization (FISH) in four cases. Immunohistochemistry of these 4 cases all showed GLI1 overexpression, which was manifested by diffuse strong positive expression of nuclei and cytoplasm of epithelial and mesenchymal components, thus providing a reliable basis for the diagnosis of gastroblastoma. Meanwhile, the fusion of the MALAT1-GLI1 gene, which indicates the activation of the Sonic hedgehog pathway in gastroblastoma, is thus expected to provide precise targeted therapy for patients with locally advanced or metastatic tumors. However, it should not be ignored that for another recently reported rare gastric tumor—plexiform fibromyxoma. The MALAT1-GLI1 fusion gene and upregulated GLI1 protein expression were also detected in some cases of this tumor, and the structure of the fusion gene was consistent with that of gastroblastoma [18]. However, the histological features of plexiform fibromyxoma lack of biphasic differentiation, which can facilitate the differentiation. Furthermore, KOO et al. [12] reported in 2021 a case of gastric submucosal tumor arising in a 17-year-old young man with Wiskott-Aldrich syndrome and a history of multiple allogeneic bone marrow transplantation and radio therapy in infancy. The histological morphology and immunohistochemical results of tumor components supported the diagnosis of gastroblastoma. However, genetic tests showed that no MALAT1-GLI1 fusion gene was found in the tumor of this patient; instead, comprehensive molecular analysis revealed a new EWSR1-CTBP1 fusion gene, with no specific changes in other genes. In this case, a 19-year-old young woman supported the diagnosis of gastroblastoma by combining the histological morphology, IHC results and the opinions of several consultation units. However, FISH results showed that the broken rearrangement of GLI1 gene and EWSR1 gene were not found in the tumor of this patient. Therefore, in view of the limited number of cases reported in gastroblastoma at home and abroad, there is still a lack of sufficient understanding of the nature of the disease and the exact gene mutation, that is, the mechanism of the occurrence and development of the disease still needs more cases and data accumulation.

Because of its histological morphology is bidirectional differentiation of epithelium and mesenchyma, gastroblastoma should be differentiated from the following diseases at diagnosis: (1) Carcinosarcoma: It has highly malignant and highly deformed cytological characteristics, and the epithelial components are squamous, adenoid, and other arrangement ways. (2) Synovial sarcoma: Rare in the stomach, mostly nodular or lobulated, cytogenetically specific t (X;18) (p11;q11), and produce SS18 (SYT)—SSX fusion gene. (3) Teratoma: The tumor is relatively diverse in composition, containing three layers of tissue, such as coelomic epithelium, neuroepithelium, and cartilage components. (4) Gastrointestinal stromal tumor (GIST): Mainly spindle or epithelioid cells, without epithelial cell differentiation, were positive for DOG1, CD34, and CD117, and SDHB expression was detected positive in this case, so SDHB-deficient GIST was excluded. In addition, PDGFR and c-kit gene mutations are often present in GIST. (5) Neuroendocrine tumors: Often present with organoid structures and are positive for Syn and CgA. (6) Glomus tumor: The tumor cells are nested or hemangiopericytomatous around the fissure blood vessels, both positive for SMA and vimentin; special PAS staining showed positive eosinophilic secretion in the cavity. (7) Plexiform fibromyxoma: Tumors present the MALAT1-GLI 1 fusion gene, but histological features lack biphasic differentiation.

Gastroblastoma is a low-grade malignancy tumor, whose biological behavior is still unclear. Among the 17 cases reported so far, most of their biological behavior are indolent, and there is usually no recurrence and metastasis after complete surgical resection, with a good prognosis. However, three cases of regional lymph nodes and distant liver and retroperitoneal metastasis, one of whom died after 7 months of follow-up [6, 10, 17]. In this case, the gastric space was found by physical examination, and the tumor was followed for 19 months after complete resection, with no tumor recurrence or metastasis.

In conclusion, gastroblastoma is a recently discovered gastric tumor with epithelial and mesenchymal biphasic differentiation. Due to the small number of cases reported in the domestic and foreign literature, the nature of this disease is not well understood, and its biological behavior is unclear. At present, most of the cases in follow-up are relatively inert clinical course, and complete surgical resection often presents with disease-free survival and a relatively good prognosis, but there is also a risk of lymph nodes and distant metastasis. Therefore, it is hoped that more cases can be found, which is expected to help medical workers deepen their understanding of the disease and avoid missed diagnosis and misdiagnosis.

## Data Availability

All data and materials are authentic and searchable, and all data and materials are available in Liuzhou Workers’ Pathology Department.

## Abbreviations

GB: Gastroblastoma
FISH: Fluorescence in situ hybridization
IHC: Immunohistochemical method
GIST: Gastrointestinal stromal tumor
CT: Computed tomography
MRI: Magnetic resonance imaging
